# Supplementary material for: Enteral Bioactive Factor Supplementation in Preterm Infants: A Systematic Review
Source: Nutrients. 2020 Sep 24;12(10):2916. doi: 10.3390/nu12102916 (PMC7598610; doi:10.3390/nu12102916)
Supplement: Supplementary file 1 [file nutrients-12-02916-s001.pdf]

Supplementary Materials

Table S1. Search strategy in MEDLINE (A), EMBASE (B), and CENTRAL (C)

A. MEDLINE

#	Searches	Results
1	exp infant, low birth weight/ or exp infant, premature/ [premature/low birth weight infant]	78657
2	exp infant, premature, diseases/ or enterocolitis, necrotizing/	46015
3	neonatal sepsis/	575
4	(((prematur* or pre-matur* or i?matur* or preterm* or pre-term* or VLBW* or ELBW* or LBW or low birth weight) adj6 (neo-nat* or neonat* or newborn* or born* or infant* or babies or child* or p?ediatr*)) or prematurity or extremely premat* or ((SGA or small-for-gestational-age) adj6 (neo-nat* or neonat* or newborn* or new* born* or infant* or babies))).tw,kf.	102740
5	((2000g or 2000-g or 1750g or 1750-g or 1500g or 1500-g or 1250g or 1250-g or 1000g or 1000-g or 750g or 750-g or 500g or 500-g or 2-000g or 2-000-g or 1-750g or 1-750-g or 1-500g or 1-500-g or 1-250g or 1-250-g or 1-000g or 1-000-g) adj7 (neo-nat* or neonat* or newborn* or new* born* or infant* or babies or birthweight* or birth weight* or BW or preterm* or pre-term* or prematur* or pre-matur*)).tw,kf.	8838
6	((infants or neonates or neo-nates or new*borns or born* or babies) adj18 (gestat* or GA or postmenstr* or post-menstr*) adj3 ("34" or "33" or "32" or "31" or "30" or "29" or "28" or "27" or "26" or "25" or "24") adj3 (week* or wk*)).tw,kf.	15263
7	((infants or neonates or neo-nates or new*borns or born* or babies) adj18 ("34" or "33" or "32" or "31" or "30" or "29" or "28" or "27" or "26" or "25" or "24") adj3 (week* or wk*) adj3 (gestat* or GA or postmenstr* or post-menstr*)).tw,kf.	15857
8	((preterm* or pre-term*) adj2 (formula or nutrit*)).tw,kf.	689
9	(necroti* adj2 enterocolit*).tw,kf.	7795
10	((neonat* or neo-nat* or new*borns or new* born* or babies) adj3 (septic?em* or sepsis or infect*)).tw,kf.	15426
11	or/1-10 [VLBW - preterm neonates]	161341
12	((controlled clinical trial or randomized controlled trial).pt. or control groups/ or double-blind method/ or random allocation/ or single-blind method/ or placebos/ or (randomi?ed or randomi?at* or randomly or random allocat* or random assign* or placebo or (controlled adj3 (study or trial))).tw,kf. or trial.ti. or ((((random* or control or intervention*) adj3 group*) or double-blind* or single-blind*).tw,kf. not (exp case-control studies/ or cohort studies/ or (retrospectiv* or case-serie*).tw,kf.))) not (("systematic review" or review or editorial).pt. or exp guidelines as topic/ or exp guideline/ or cochrane.jw. or (review or guidelin* or consensus or editorial or reply).ti.)	1417561
13	11 and 12 [RCTs on preterm neonates]	13366
14	((exp animals/ or exp veterinary medicine/ or exp animal diseases/ or cattle/ or (cow or cows).ti.) not (humans/ or human*.ti.)) or (animal* or veterinar*).jw. or exp pregnancy, animal/ or exp rodentia/ or exp animals, genetically modified/ or exp animals, laboratory/ or animals, newborn/ or (primates or ape or apes or monkey* or baboon* or macaq* or pig or pigs or piglet* or goat or goats* or sheep or lamb or lambs or ovine or cattle or horse or horses or mare or calve or calves or dog or dogs or canine or bitch* or cat or cats or feline or rodent* or rabbit* or mice or mouse or murine* or rat or rats or chick* or broiler or frog or frogs or C57BL* or Balb-c or Balbc or wistar or sprague or dawley or dam or dams or pups or pup or ewe or ewes or sow or sows).ti,kf.	5736995
15	13 not 14 [human RCTs on preterm neonates]	12267
16	limit 15 to yr="2000 -Current" [human RCTs on preterm infants >2000]	8325

17	(pregestat* or preconcept* or conception or prepregnan* or pregnan* or mid-gestat* or midgestat* or gravidity or multigravid* or multigestat* or during gestation or placenta* or preterm ruptur* or preterm birth* or preterm labo?r or PPROM or PROM or f?etal or f?etus* or antenat* or ante-nat* or prenat* or pre-nat* or antepart* or ante-part* or peripart* or peri-part* or postpart* or post-part* or perinat* or peri-nat* or pregestat* or preeclamp* or eclamp* or HELPP or ((maternal or gestation*) adj3 (diabet* or hyperten*)) or HIV* or AIDS or tuberculos* or ((mother* or women or matern*) not (infant* or neonat* or neo-nat* or new*-born* or new*born*)) or ((parenteral* or intravenous* or intra-venous* or iv) not (enteral* or enteric or oral*))).ti. or (((maternal or mother* or women or antenat* or ante-nat* or prenat* or pre-nat*) adj6 (suppl* or diet* or nutrition)) not ((infant* or neonat* or neo-nat* or newborn* or new* born*) adj6 (suppl* or diet* or nutrition))).tw,kf. [TW exclusion pregnant/maternal/HIV/parenteral]	1246332
18	16 not 17 [human RCTs on preterm infants >2000 (not antenatal supplementation)]	6431
19	dietary supplements/	55931
20	prebiotics/ or synbiotics/	2858
21	exp fructans/ or galactans/ or glucans/ or beta-glucans/ or glycosaminoglycans/ or oligosaccharides/ or pectins/	72971
22	colostrum/ and (cattle/ or enteral nutrition/ or administration, oral/ or administration, buccal/ or oropharynx/ or infant formula/ or food, fortified/)	2648
23	caseins/ or exp whey proteins/	29052
24	glycoproteins/ad, pk, pd, tu or exp mucins/ad, pk, pd, tu	5599
25	carotenoids/ or beta carotene/ or lycopene/ or exp xanthophylls/	26464
26	ascorbic acid/ad, tu, pk, pd or tocopherols/ad, tu, pk, pd or vitamin a/ad, tu, pk, pd or vitamin e/ad, tu, pk, pd or alpha-tocopherol/ad, tu, pk, pd or gamma-tocopherol/ad, tu, pk, pd	43497
27	trace elements/ad, tu, pk, pd or copper/ad, tu, pk, pd or iodine/ad, tu, pk, pd or manganese/ad, tu, pk, pd or molybdenum/ad, tu, pk, pd or selenic acid/ad, tu, pk, pd or selenious acid/ad, tu, pk, pd or selenium/ad, tu, pk, pd or sodium selenite/ad, tu, pk, pd or zinc/ad, tu, pk, pd or zinc sulfate/ad, tu, pk, pd	48638
28	(amino acids, essential/ad, tu, pd, pk or arginine/ad, tu, pd, pk or histidine/ad, tu, pd, pk or isoleucine/ad, tu, pd, pk or leucine/ad, tu, pd, pk or lysine/ad, tu, pd, pk or methionine/ad, tu, pd, pk or phenylalanine/ad, tu, pd, pk or threonine/ad, tu, pd, pk or tryptophan/ad, tu, pd, pk or valine/ad, tu, pd, pk or tyrosine/ad, tu, pk, pd or asparagine/ad, tu, pk, pd or glutamine/ad, tu, pk, pd or glycine/ad, tu, pk, pd or cysteine/ad, tu, pk, pd or acetylcysteine/ad, tu, pk, pd or ornithine/ad, tu, pk, pd or serine/ad, tu, pk, pd or proline/ad, tu, pk, pd) not (*pain/dt or *ibuprofen/ad, tu)	77350
29	exp carboxylic ester hydrolases/ad, tu, pd, pk or lipase/ or sterol esterase/ or exp amylases/ or lactoperoxidase/ad, tu	47086
30	(recombinant proteins/ or exp antioxidants/ad, tu, pk or exp superoxide dismutase/ad, tu, pk or catalase/ad, tu, pk or glutathione peroxidase/ad, tu, pk or muramidase/ad, tu, pk or (recombinant or rec or rh-epo or r-HuEPO or rhepo).tw,kf.) and (enteral nutrition/ or infant formula/ or bottle feeding/ or infant nutritional physiological phenomena/ or infant food/ or administration, oral/ or (enteral* or feed* or fed or formula or oral*).tw,kf.)	29197
31	(fatty acids/ad, tu or fatty acids, unsaturated/ad, tu or exp fatty acids, essential/ad, tu, pd, pk or fatty acids, monounsaturated/ or exp fatty acids, omega-3/ or exp docosahexaenoic acids/ or exp fatty acids, omega-6/) not (administration, cutaneous/ or skin absorption/)	59563
32	triglycerides/ad, tu or (exp membrane lipids/ad, tu not (surfact* or galactogog*).mp.)	22549
33	(triglycerides/ or (exp membrane lipids/ not (surfact* or galactogog*).mp.)) and enteral nutrition/	237

34	(exp "intercellular signaling peptides and proteins"/ad, tu or (exp hormones/ad, tu not oxytocics/ad, tu) or exp hormone replacement therapy/ or insulin/pd, pk or exp immunologic factors/ad, tu or exp carrier proteins/ad, tu) and (enteral nutrition/ or infant formula/ or bottle feeding/ or milk,human/ or infant food/ or infant nutritional physiological phenomena/ or infant food/ or enteral*.tw,kf.)	1445
35	melatonin/ or exp adipokines/ad, tu, pd or GPI-linked proteins/ad, tu, pd or osteopontin/ad, tu, pd or nicotinamide phosphoribosyltransferase/ad, tu or nucb2 protein human.rn. or exp cytokines/ai or exp receptors, cytokine/ai	64819
36	(((supplementat* or supplementing or supplemented) not (suppl* adj3 oxygen*)) or unsupplement* or un-supplem* or nonsupplem* or non-supplem* or ((diet* or nutri* or enteral* or enteric or oral* or os or oro* or nasogastr* or experimental or enhanc* or enrich* or intake or added or contain* or whey or milk or breastmilk or formula or formulas or powder) adj2 (supplements or supplement))).tw,kf.	213679
37	(((added or adding or (addition not "in-addition") or additions) adj6 (formula* or powder or milk or breastmilk or enteral* or enteric or feed* or nutrition or diet or diets)) or ((experimental or content* or composit* or free or deplet*) adj2 formula*) or formula*-containing or "formula* without").tw,kf.	26434
38	(((enteral* or enteric) adj2 (administ* or enrich* or dose or doses or daily)) or ((enteral* or enteric) adj1 (given or intervent*))).tw,kf.	1820
39	(((oral* not oral glucos*) or os or oro* or nasogastr* or intragastric or intra-gastr* or enteral* or enteric or fortif* or enrich* or suppl* or replac*) adj6 (enzym* or amino acid* or aminoacid* or antioxidant* or anti-oxidant* or bioactiv* factors)).tw,kf.	36385
40	(prebiot* or pre-biot* or synbiot* or syn-biot* or oligosacch* or oligo-sacch* or fructooligo* or galactooligo* or xylooligo* or HMO or HMOs or SCGOS* or LCFOS* or GOS or FOS or (inulin* not (inulin* adj2 clear*)) or fructan* or galactan* or pectin* or AOS or pAOS or betaglucan* or glucan*).tw,kf.	120346
41	(((bovine or oro* or oral* or buccal* or enteral* or enteric) adj6 colostrum) or forticolos or precolos).tw,kf.	1075
42	(whey or casein* or gl*comacropeptid* or gl*co-macropept* or GMP or lact*albumin* or lacto-albumin* or lact*ferrin* or lacto-ferrin* or bLF or bovine-LF or TLF or LIFT or ELFIN or Talactoferrin* or lactadherin* or MFGM* or lactogammaglobulin* or lacto-gammaglobulin or milk-fat-globulin-membran*).tw,kf.	74290
43	(caroten* or betacarot* or lycopen* or xanthophyl* or lutein or zeaxant?in* or zea-xant?in* or cryptoxant?in* or crypto-xant?in*).tw,kf.	36648
44	(vitamin-A or vit-A or vitamin-C or vit-C or vitamin-E or vit-E).tw,kf.	69748
45	(((oral* not oral glucos*) or os or oro* or nasogastr* or intragastric or intra-gastr* or enteral* or enteric or fortif* or enrich* or suppl* or replac*) adj6 (vit or vitamin* or multivitamin* or retinol or ascorbic* or ascorbat* or tocopherol* or alphatocopherol* or tocotrienol*)).tw,kf.	40603
46	((oral* or os or oro* or nasogastr* or enteral* or enteric or fed or feed* or nutrition* or diet or diets or formula or formulas or whey or fortif* or enrich* or added or adding or additions or suppl* or mix*) adj6 (trace element* or multimicronutrien* or micronutrient* or zinc or Zn* or selen* or copper or mangan* or molybden* or iodine)).tw,kf.	36766
47	(acetylcysteine or Nacetylcysteine or NAC or cysteine or cystine or (CYS not Cys-C) or ((oral* or os or oro* or nasogastr* or enteral* or enteric or fed or feed* or nutrition* or diet or diets or formula or formulas or powder or whey or fortif* or enrich* or added or adding or additions or administration or dose or doses or content or composit* or contain* or suppl* or mix*) adj6 (essential amino acid* or arginin* or histidin* or isoleucin* or leucin* or (lysin* not (ibuprof* adj3 lysin*)) or (methionin* not S-adenosyl-L-methionin*) or phenylalanin* or fenylalanin* or threonin* or phosphot?reonin* or tryptophan* or tyrosine or valine or asparagine or glutamin* or glycine or levocarnitin* or carnitin*))).tw,kf.	207237

48	((recomb* adj10 lipase*) or r-lipas* or rh-lipas* or bucelipas* or BSSL* or rhBSSL* or ((oral* or os or oro* or nasogastr* or enteral* or enteric or fed or feed* or nutrition* or diet or diets or formula or formulas or powder or whey or fortif* or enrich* or added or adding or additions or administration or dose or doses or content or composit* or contain* or suppl* or mix*) adj6 (lipas* or esteras* or carboxylesteras* or glycosidas* or protease* or amylas* or acetylhydrolas* or (acetyl adj3 hydrolas*) or esteras* or catalas* or superoxide dismutase* or rhSOD* or CuZNSOD* or SOD or reductas* or oxi*reductas* or peroxidas* or lact*peroxida* or transferas* or lysozym* or muramidas*))).tw,kf.	41672
49	(polyunsaturat* or poly-unsaturat* or monounsaturat* or mono-unsaturat* or MUFA or MUFAs or PUFA or PUFAs or LCPUFA* or LCP or LCPs or docosahex?eno* or DHA or eicosapent?en* or icosapent?en* or EPA or omega-3* or omega-6* or omega3* or omega6*).tw,kf. or ((n3 or n6 or n-3 or n-6).tw,kf. and (fatty acid* or LA or ALA).mp.)	77717
50	(linolenic or linolenate* or alphalinolen* or gammalinolen* or GLA or DGLA or arachidon* or ARA).tw,kf. not (cutan* or transcutan* or subcutan*).ti.	67330
51	((oral* or os or oro* or nasogastr* or enteral* or enteric or fed or feed* or nutrition* or diet or diets or formula or formulas or powder or whey or fortif* or enrich* or added or adding or additions or content or composit* or contain* or suppl* or mix*) adj6 fatty acid*).tw,kf. not (cutan* or transcutan* or subcutan*).ti.	47713
52	(melatonin* or l?evothyroxin* or levoxin* or l?evo-thyroxin* or L-thyroxin* or LT4 or LT-4).tw,kf.	31529
53	(((oral* not oral glucos*) or os or oro* or nasogastr* or intragastric or intra-gastr* or enteral* or enteric or fortif* or enrich* or suppl* or replac*) adj9 (recombinant or hormon* or cytokin* or chemokin* or GLP or glucagon* or GLP1 or GLP2 or PYY or peptid*-YY or leptin* or adipokin* or adiponect* or ADPN or adipofibrokin* or adipocytokin* or adipoq* or resistin* or FIZZ3 or FIZZ-3 or inflammator*-zone-3-protein* or ADSF or RELM or visfatin* or VISF or NAMPT* or NAmPRTas* or ((NMN or nicotinamid* or pyrophosphat*) adj2 (pyrophosphorylas* or phosphoribosyltransferas* or transphosphoribosylas*)) or PBEF or PBEF1 or (pre-B adj2 colony enhancing factor*) or obestatin* or ghrelin* or Ppghrelin* or apelin* or APJ or APE-12 or APE12 or APE-13 or APE13 or APE-36 or APE36 or APLN* or estradiol* or oestradiol* or estriol or oestriol or estrogen* or oestrogen* or estron* or progest* or hydroxyprogest* or calcitonin* or calcitrin* or somatostatin* or prolactin* or cortisol or cortisone or hydrocortison* or thyro* or thyroxin* or T4 or T-4 or liothyronin* or T3 or T-3 or insulin* or carrier-protein* or binding protein* or nucleobindin* or NUCB2 or nesfatin* or NEFA or IGFB* or calcium-binding or calprotectin* or calgranulin* or 27E10 or CABP-P8 or MRP8 or MRP-8 or 60B8* or CABP-P14 or MRP14 or MRP-14 or S100* or S-100A* or S-100B* or dermatopontin* or (dpt adj3 protein) or discoidin* or growth factor* or somatomedin* or IGF or IGF1 or IGF2 or IGFB* or rhIGF* or EGF or urogastron* or TGF* or HGF or hepatopoietin* or neurotrophic factor* or NGF* or BDNF* or GDN* or VEGF* or sVEGF* or vasculotropin* or ANG or ANG1 or ANG2 or angiopoietin* or FGF* or colony-stimulating factor* or CSF* or GCSF* or GMCSF* or EPO or rhEPO or rHuEPO or HuEPO or erythropoietin* or epoietin* or darpoetin* or interleukin* or lymphokin* or IL or IL7 or IL8 or CXCL* or C-X-C or CXC or MCP-1 or MCP1 or sCXCL* or IL10 or CSIF-10 or CSIF10 or TNF* or tumo?r-necrosis-factor* or MIF or migration inhibition factor* or immunoglob* or Ig or IgA or sIgA or IgG* or IgM*)).tw,kf.	109953
54	or/19-53 [dietary supplements; bioactive factors]	1323741
55	18 and 54 [human RCTs on preterm infants + enteral supplementation]	734
56	remove duplicates from 55 [human RCTs on preterm infants + enteral supplementation - deduplicated]	732

B. EMBASE

#	Searches	Results
1	newborn disease/ or exp low birth weight/ or newborn sepsis/ or neonatal respiratory distress syndrome/ or prematurity/ or retrolental fibroplasia/ [premature/low birth weight infant/newborn disease]	201900
2	necrotizing enterocolitis/	11368
3	(((prematur* or pre-matur* or i?matur* or preterm* or pre-term* or VLBW* or ELBW* or LBW or low birth weight) adj6 (neo-nat* or neonat* or newborn* or born* or infant* or babies or child* or p?ediatr*)) or prematurity or extremely premat* or ((SGA or small-for-gestational-age) adj6 (neo-nat* or neonat* or newborn* or new* born* or infant* or babies))).tw,kw.	141996
4	((2000g or 2000-g or 1750g or 1750-g or 1500g or 1500-g or 1250g or 1250-g or 1000g or 1000-g or 750g or 750-g or 500g or 500-g or 2-000g or 2-000-g or 1-750g or 1-750-g or 1-500g or 1-500-g or 1-250g or 1-250-g or 1-000g or 1-000-g) adj7 (neo-nat* or neonat* or newborn* or new* born* or infant* or babies or birthweight* or birth weight* or BW or preterm* or pre-term* or prematur* or pre-matur*)).tw,kw.	11917
5	((infants or neonates or neo-nates or new*borns or born* or babies) adj18 (gestat* or GA or postmenstr* or post-menstr*) adj3 ("34" or "33" or "32" or "31" or "30" or "29" or "28" or "27" or "26" or "25" or "24") adj3 (week* or wk*)).tw,kw.	22038
6	((infants or neonates or neo-nates or new*borns or born* or babies) adj18 ("34" or "33" or "32" or "31" or "30" or "29" or "28" or "27" or "26" or "25" or "24") adj3 (week* or wk*) adj3 (gestat* or GA or postmenstr* or post-menstr*)).tw,kw.	22667
7	((preterm* or pre-term*) adj2 (formula or nutrit*)).tw,kw.	929
8	(necroti* adj2 enterocolit*).tw,kw.	10824
9	((neonat* or neo-nat* or new*borns or new* born* or babies) adj3 (septic?em* or sepsis or infect*)).tw,kw.	21492
10	or/1-9 [VLBW - preterm neonates]	261231
11	(randomized controlled trial/ or controlled clinical trial/ or control group/ or double blind procedure/ or single blind procedure/ or randomization/ or placebo/ or (randomi?ed or randomi?at* or randomly or random allocat* or random assign* or placebo or (controlled adj3 (study or trial))).tw,kw. or trial.ti. or ((((random* or control or intervention*) adj3 group*) or double-blind* or single-blind*).tw,kw. not (exp case control study/ or case report/ or retrospective study/ or (retrospectiv* or case-serie*).tw,kw.))) not ((review or editorial or note or short survey).pt. or editorial/ or review/ or short survey/ or systematic review/ or practice guideline/ or consensus development/ or cochrane.jw. or (review or guidelin* or consensus or editorial or reply).ti.) [RCT-filter]	2063586
12	10 and 11 [RCTs on preterm neonates]	20900
13	((exp animal/ or animal experiment/ or exp animal model/ or nonhuman/ or exp female animal/ or exp domestic cattle/ or (cow or cows).ti.) not (human/ or human*.ti.)) or (animal* or veterinar*).jw. or exp rodent/ or exp experimental animal/ or (primates or ape or apes or monkey* or baboon* or macaq* or pig or pigs or piglet* or goat or goats* or sheep or lamb or lambs or ovine or cattle or horse or horses or mare or calve or calves or dog or dogs or canine or bitch* or cat or cats or feline or rodent* or rabbit* or mice or mouse or murine* or rat or rats or chick* or broiler or frog or frogs or C57BL* or Balb-c or Balbc or wistar or sprague or dawley or dam or dams or pups or pup or ewe or ewes or sow or sows).ti,kw. [animal filter]	8292276
14	12 not 13 [RCTs on preterm human neonates]	19012
15	limit 14 to yr="2000 -Current" [human RCTs on preterm infants >2000]	14707

16	(pregestat* or preconcept* or conception or prepregnan* or pregnan* or mid-gestat* or midgestat* or gravidity or multigravid* or multigestat* or during gestation or placenta* or preterm ruptur* or preterm birth* or preterm labo?r or PPROM or PROM or f?etal or f?etus* or antenat* or ante-nat* or prenat* or pre-nat* or antepart* or ante-part* or peripart* or peri-part* or postpart* or post-part* or perinat* or peri-nat* or pregestat* or preeclamp* or eclamp* or HELPP or ((maternal or gestation*) adj3 (diabet* or hyperten*)) or HIV* or AIDS or tuberculos* or ((mother* or women or matern*) not (infant* or neonat* or neo-nat* or new*-born* or new*born*)) or ((parenteral* or intravenous* or intra-venous* or iv) not (enteral* or enteric or oral*))).ti. or (((maternal or mother* or women or antenat* or ante-nat* or prenat* or pre-nat*) adj6 (suppl* or diet* or nutrition)) not ((infant* or neonat* or neo-nat* or newborn* or new* born*) adj6 (suppl* or diet* or nutrition))).tw,kw. [TW exclusion pregnant/maternal/HIV/parenteral]	1563480
17	15 not 16	10376
18	dietary supplement/ or diet supplementation/ or mineral supplementation/ or vitamin supplementation/ or ((supplementation/ or exp nutrition supplement/) and ((ig or po).fs. or enteric feeding/ or exp enteral drug administration/ or artificial milk/ or breast milk/ or nutritional support/ or dietary intake/ or diet/ or diet therapy/ or (enteric or enteral).tw,kw. or oral*.ti.))	139629
19	oligosaccharide/ or fructose oligosaccharide/ or galactose oligosaccharide/ or prebiotic agent/ or synbiotic agent/	34708
20	beta 1,3 glucan/ or beta 1,6 glucan/ or fructan/ or galactan/ or glucan/ or pectin/ or inulin/ct, ad, cb, cm, do, dt, ig, po or glycosaminoglycans/ct, ad, cb, cm, do, dt, ig, po	22251
21	glycoprotein/ct, ad, cb, cm, do, dt, ig, po or mucin/ct, ad, cb, cm, do, dt, ig, po or mucin 2/ct, ad, cb, cm, do, dt, ig, po or mucin 1/ct, ad, cb, cm, do, dt, ig, po or mucin 3/ct, ad, cb, cm, do, dt, ig, po or mucin 4/ct, ad, cb, cm, do, dt, ig, po or mucin 5ac/ct, ad, cb, cm, do, dt, ig, po or mucin 5b/ct, ad, cb, cm, do, dt, ig, po or mucin 6/ct, ad, cb, cm, do, dt, ig, po or mucin 7/ct, ad, cb, cm, do, dt, ig, po or mucin 8/ct, ad, cb, cm, do, dt, ig, po	1067
22	colostrum/po, ig or (colostrum/ and (bovine/ or enteric feeding/ or exp enteral drug administration/ or oropharynx/ or infant feeding/ or artificial milk/ or supplements/ or supplementation/))	1025
23	casein/ or whey/ or whey protein/ or lactalbumin/ or lactoglobulin/ or lactoferrin/ or talactoferrin/	40789
24	carotenoid/ or beta carotene/ or beta cryptoxanthin/ or canthaxanthin/ or cryptoxanthin/ or lycopene/ or neoxanthin/ or xanthophyll/ or zeaxanthin/	45429
25	retinol/ct, ad, cb, cm, do, po or tocopherol/ct, ad, cb, cm, do, po or alpha tocopherol/ct, ad, cb, cm, do, po or gamma tocopherol/ct, ad, cb, cm, do, po or ascorbic acid/ct, ad, cb, cm, do, dt, ig, po, li or ((retinol/ or tocopherol/ or alpha tocopherol/ or gamma tocopherol/ or ascorbic acid/) and (enteric feeding/ or exp enteral drug administration/)) or *retinol/ or *tocopherol/ or *alpha tocopherol/ or *gamma tocopherol/ or *ascorbic acid/	99720
26	trace element/ct, ad, cb, cm, do, dt, ig, po or zinc/ct, ad, cb, cm, do, dt, ig, po or gluconate zinc/ or zinc sulfate/ or zinc derivative/ or selenium/ct, ad, cb, cm, do, dt, ig, po or selenate/ or sodium selenite/ or copper/ct, ad, cb, cm, do, dt, ig, po or manganese/ct, ad, cb, cm, do, dt, ig, po or molybdenum/ct, ad, cb, cm, do, dt, ig, po or iodine/ct, ad, cb, cm, do, dt, ig, po or sodium iodide/ct, ad, cb, cm, do, dt, ig, po or (*trace element/ not trace element/ec) or *selenium/ or (*zinc/ not *zinc/ec) or ((zinc/ or selenium/ or copper/ or manganese/ or molybdenum/ or iodine/ or sodium iodide/) and (enteric feeding/ or exp enteral drug administration/ or dietary intake/ or infant nutrition/ or artificial milk/ or supplementation/))	108654

27	exp essential amino acid/ct, ad, cb, cm, do, dt, ig, po or *amino acid/ad, dt, po or tyrosine/ct, ad, cb, cm, do, dt, ig, po or proline/ct, ad, cb, cm, do, dt, ig, po or tyrosine/ct, ad, cb, cm, do, dt, ig, po or asparagine/ct, ad, cb, cm, do, dt, ig, po or glutamine/ct, ad, cb, cm, do, dt, ig, po or glycine/ct, ad, cb, cm, do, dt, ig, po or acetylcysteine/ct, ad, cb, cm, do, dt, ig, po or cysteine/ct, ad, cb, cm, do, dt, ig, po or ornithine/ct, ad, cb, cm, do, dt, ig, po or serine/ct, ad, cb, cm, do, dt, ig, po or carnitine/ct, ad, cb, cm, do, dt, ig, po or (*glutamine/ not glutamine/ec) or (*acetylcysteine/ not acetylcysteine/ec) or (*cysteine/ not cysteine/ec) or ((exp essential amino acid/ or proline/ or tyrosine/ or asparagine/ or glutamine/ or glycine/ or acetylcysteine/ or cysteine/ or ornithine/ or serine/ or carnitine/) and (enteric feeding/ or exp enteral drug administration/ or dietary intake/ or infant nutrition/ or artificial milk/ or supplementation/))	70691
28	(bucelipase alfa/ or esterase/ct, ad, cb, cm, do, dt, ig, po or acid lipase/ct, ad, cb, cm, do, dt, ig, po or triacylglycerol lipase/ct, ad, cb, cm, do, dt, ig, po or exp esterase/ad, tu, po or carboxylesterase/ct, ad, do, dt or amylase/ct, ad, cb, cm, do, dt, ig, po or exp glycosidase/ad, tu, po or exp protease/po or lactoperoxidase/ct, ad, cb, cm, do, dt, ig, po or glycosidase/ct, ad, cb, cm, do, dt, ig, po or exp lysozyme/ct, ad, cb, cm, do, dt, ig, po or 1 alkyl 2 acetylglycerophosphocholine esterase/ or oxidoreductase/ct, ad, cb, cm, do, dt, ig, po or catalase/ct, ad, cb, cm, do, dt, ig, po or superoxide dismutase/ct, ad, cb, cm, do, dt, ig, po or ((esterase/ or acid lipase/ or triacylglycerol lipase/ or carboxylesterase/ or glycosidase/ or amylase/ or exp lysozyme/ or oxidoreductase/ or catalase/ or superoxide dismutase/) and (enteric feeding/ or exp enteral drug administration/ or dietary intake/ or infant nutrition/ or artificial milk/ or supplementation/))) not (superoxide dismutase/ and (lung surfactant/ or surfactant/ or superoxide dismutase/tr))	13544
29	(exp essential fatty acid/ or exp medium chain fatty acid/ or exp unsaturated fatty acid/ or medium chain triacylglycerol/) not (exp unsaturated fatty acid/tp or (cutaneous or topical* or transcutan* or subcutan* or massag*).ti.)	172347
30	exp phospholipid/ct, ad, cb, cm, do, ig, po or sphingolipid/ or sphingomyelin/ or phosphatidylcholine/ct, ad, do, ig, po or (phosphatidylcholine/ not (phosphatidylcholine/ec, tp or (surfact* or galactogog*).ti,hw.)) or lysophospholipid/ or membrane phospholipid/	58770
31	melatonin/ or levothyroxine/ or obestatin/ or exp thyromimetic agent/ig, po or hydrocortisone/ig, po or hydrocortisone sodium succinate/ig, po or exp sex hormone/ig, po or exp "peptides and proteins"/ig, po or ((hormonal therapy/ or exp hormone substitution/ or exp thyromimetic agent/ct, ad, cb, cm, do or hydrocortisone/ct, ad, cm, do or hydrocortisone sodium succinate/ct, ad, cm, do or exp "peptides and proteins"/ct, ad, cb, cm, do or recombinant protein/ or recombinant enzyme/ or recombinant leptin/ or exp recombinant growth factor/ or exp *recombinant hormone/ or exp sex hormone/ct, ad, cm, do or recombinant somatomedin C/ or exp recombinant cytokine/ or (exp *peptide hormone/ not exp peptide hormone/ec) or (*hydrocortisone/ not hydrocortisone/ec, iv) or (*hydrocortisone sodium succinate/ not hydrocortisone sodium succinate/ec, iv) or (exp *sex hormone/ not exp sex hormone/ec) or (exp *cytokine/ not exp cytokine/ec) or (exp *growth factor/ not exp growth factor/ec, iv, sc) or (exp *immunoglobulin/ not exp *immunoglobulin/ec, iv, sc)) and (enteric feeding/ or exp enteral drug administration/ or dietary intake/ or infant nutrition/ or artificial milk/ or supplementation/ or (enteral* or enteric).tw,kw.))	151384
32	(((supplementat* or supplementing or supplemented) not (suppl* adj3 oxygen*)) or unsupplement* or un-supplem* or nonsupplem* or non-supplem* or ((diet* or nutri* or enteral* or enteric or oral* or os or oro* or nasogastr* or experimental or enhanc* or enrich* or intake or added or contain* or whey or milk or breastmilk or formula or formulas or powder) adj2 (supplements or supplement))).tw,kw.	284765
33	(((added or adding or (addition not "in-addition") or additions) adj6 (formula* or powder or milk or breastmilk or enteral* or enteric or feed* or nutrition or diet or diets))	36025

	or ((experimental or content* or composit* or free or deplet*) adj2 formula*) or formula*-containing or "formula* without").tw,kw.	
34	(((enteral* or enteric) adj2 (administ* or enrich* or dose or doses or daily)) or ((enteral* or enteric) adj1 (given or intervent*))).tw,kw.	2430
35	(((oral* not oral glucos*) or os or oro* or nasogastr* or intragastric or intra-gastr* or enteral* or enteric or fortif* or enrich* or suppl* or replac*) adj6 (enzym* or amino acid* or aminoacid* or antioxidant* or anti-oxidant* or bioactiv* factors)).tw,kw.	48073
36	(prebiot* or pre-biot* or synbiot* or syn-biot* or oligosacch* or oligo-sacch* or fructooligo* or galactooligo* or xylooligo* or HMO or HMOs or SCGOS* or LCFOS* or GOS or FOS or (inulin* not (inulin* adj2 clear*)) or fructan* or galactan* or pectin* or AOS or pAOS or betaglucan* or glucan*).tw,kw.	143033
37	(((bovine or oro* or oral* or buccal* or enteral* or enteric) adj6 colostrum) or forticolos or precolos).tw,kw.	1262
38	(whey or casein* or gl*comacropeptid* or gl*co-macropept* or GMP or lact*albumin* or lacto-albumin* or lact*ferrin* or lacto-ferrin* or bLF or bovine-LF or TLF or LIFT or ELFIN or Talactoferrin* or lactadherin* or MFGM* or lactogammaglobulin* or lacto-gammaglobulin or milk-fat-globulin-membran*).tw,kw.	95835
39	(caroten* or betacarot* or lycopen* or xanthophyl* or lutein or zeaxant?in* or zea-xant?in* or cryptoxant?in* or crypto-xant?in*).tw,kw.	43220
40	(vitamin-A or vit-A or vitamin-C or vit-C or vitamin-E or vit-E).tw,kw.	90675
41	(((oral* not oral glucos*) or os or oro* or nasogastr* or intragastric or intra-gastr* or enteral* or enteric or fortif* or enrich* or suppl* or replac*) adj6 (vit or vitamin* or multivitamin* or retinol or ascorbic* or ascorbat* or tocopherol* or alphatocopherol* or tocotrienol*)).tw,kw.	60572
42	((oral* or os or oro* or nasogastr* or enteral* or enteric or fed or feed* or nutrition* or diet or diets or formula or formulas or whey or fortif* or enrich* or added or adding or additions or suppl* or mix*) adj6 (trace element* or multimicronutrien* or micronutrient* or zinc or Zn* or selen* or copper or mangan* or molybden* or iodine)).tw,kw.	46819
43	(acetylcysteine or Nacetylcysteine or NAC or cysteine or cystine or (CYS not Cys-C) or ((oral* or os or oro* or nasogastr* or enteral* or enteric or fed or feed* or nutrition* or diet or diets or formula or formulas or powder or whey or fortif* or enrich* or added or adding or additions or administration or dose or doses or content or composit* or contain* or suppl* or mix*) adj6 (essential amino acid* or arginin* or histidin* or isoleucin* or leucin* or (lysin* not (ibuprof* adj3 lysin*)) or (methionin* not S-adenosyl-L-methionin*) or phenylalanin* or fenylalanin* or threonin* or phosphot?reonin* or tryptophan* or tyrosine or valine or asparagine or glutamin* or glycine or levocarnitin* or carnitin*))).tw,kw.	255877
44	((recomb* adj10 lipase*) or r-lipas* or rh-lipas* or bucelipas* or BSSL* or rhBSSL* or ((oral* or os or oro* or nasogastr* or enteral* or enteric or fed or feed* or nutrition* or diet or diets or formula or formulas or powder or whey or fortif* or enrich* or added or adding or additions or administration or dose or doses or content or composit* or contain* or suppl* or mix*) adj6 (lipas* or esteras* or carboxylesteras* or glycosidas* or protease* or amylas* or acetylhydrolas* or (acetyl adj3 hydrolas*) or esteras* or catalas* or superoxide dismutase* or rhSOD* or CuZNSOD* or SOD or reductas* or oxi*reductas* or peroxidas* or lact*peroxida* or transferas* or lysozym* or muramidas*))).tw,kw,dq.	50790
45	(polyunsaturat* or poly-unsaturat* or monounsaturat* or mono-unsaturat* or MUFA or MUFAs or PUFA or PUFAs or LCPUFA* or LCP or LCPs or docosahex?eno* or DHA or eicosapent?en* or icosapent?en* or EPA or omega-3* or omega-6* or omega3* or omega6*).tw,kw. or ((n3 or n6 or n-3 or n-6).tw,kw. and (fatty acid* or LA or ALA).mp.)	99620

46	(linolenic or linolenate* or alphalinolen* or gammalinolen* or GLA or DGLA or arachidon* or ARA).tw,kw. not (cutan* or transcutan* or subcutan*).ti.	85356
47	((oral* or os or oro* or nasogastr* or enteral* or enteric or fed or feed* or nutrition* or diet or diets or formula or formulas or powder or whey or fortif* or enrich* or added or adding or additions or content or composit* or contain* or suppl* or mix*) adj6 fatty acid*).tw,kw. not (cutan* or transcutan* or subcutan*).ti.	58704
48	(melatonin* or l?evothyroxin* or levoxin* or l?evo-thyroxin* or L-thyroxin* or LT4 or LT-4).tw,kw.	43212
49	(((oral* not oral glucos*) or os or oro* or nasogastr* or intragastric or intra-gastr* or enteral* or enteric or fortif* or enrich* or suppl* or replac*) adj9 (recombinant or hormon* or cytokin* or chemokin* or GLP or glucagon* or GLP1 or GLP2 or PYY or peptid*-YY or leptin* or adipokin* or adiponect* or ADPN or adipofibrokin* or adipocytokin* or adipoq* or resistin* or FIZZ3 or FIZZ-3 or inflammator*-zone-3-protein* or ADSF or RELM or visfatin* or VISF or NAMPT* or NAmPRTas* or ((NMN or nicotinamid* or pyrophosphat*) adj2 (pyrophosphorylas* or phosphoribosyltransferas* or transphosphoribosylas*)) or PBEF or PBEF1 or (pre-B adj2 colony enhancing factor*) or obestatin* or ghrelin* or Ppghrelin* or apelin* or APJ or APE-12 or APE12 or APE-13 or APE13 or APE-36 or APE36 or APLN* or estradiol* or oestradiol* or estriol or oestriol or estrogen* or oestrogen* or estron* or progest* or hydroxyprogest* or calcitonin* or calcitrin* or somatostatin* or prolactin* or cortisol or cortisone or hydrocortison* or thyro* or thyroxin* or T4 or T-4 or liothyronin* or T3 or T-3 or insulin* or carrier-protein* or binding protein* or nucleobindin* or NUCB2 or nesfatin* or NEFA or IGFB* or calcium-binding or calprotectin* or calgranulin* or 27E10 or CABP-P8 or MRP8 or MRP-8 or 60B8* or CABP-P14 or MRP14 or MRP-14 or S100* or S-100A* or S-100B* or dermatopontin* or (dpt adj3 protein) or discoidin* or growth factor* or somatomedin* or IGF or IGF1 or IGF2 or IGFB* or rhIGF* or EGF or urogastron* or TGF* or HGF or hepatopoietin* or neurotrophic factor* or NGF* or BDNF* or GDN* or VEGF* or sVEGF* or vasculotropin* or ANG or ANG1 or ANG2 or angiopoietin* or FGF* or colony-stimulating factor* or CSF* or GCSF* or GMCSF* or EPO or rhEPO or rHuEPO or HuEPO or erythropoietin* or epoietin* or darpoetin* or interleukin* or lymphokin* or IL or IL7 or IL8 or CXCL* or C-X-C or CXC or MCP-1 or MCP1 or sCXCL* or IL10 or CSIF-10 or CSIF10 or TNF* or tumo?r-necrosis-factor* or MIF or migration inhibition factor* or immunoglob* or Ig or IgA or sIgA or IgG* or IgM*)).tw,kw.	159399
50	or/18-49 [dietary supplements; bioactive factors]	1749778
51	17 and 50 [human RCTs on preterm infants + enteral supplementation]	1230
52	remove duplicates from 51 [human RCTs on preterm infants + enteral supplementation -deduplicated]	1202
53	52 not medline.cr. [human RCTs on preterm infants + enteral supplementation -deduplicated -embase records only]	1128

C. CENTRAL

The search in CENTRAL was splitted in three parts. The hits were merged and duplicates were removed. The number of unique hits was 1046.

Part 1

#	Searches	Results
#1	(((prematur* or pre-matur* or immatur* or preterm* or pre-term* or VLBW* or ELBW* or LBW or low-birth-weight) near/6 (neo-nat* or neonat* or newborn* or born* or infant* or babies or child* or pediatr* or paediatr*)) or prematurity or ((SGA or small-for-gestational-age) near/6 (neo-nat* or neonat* or newborn* or (new* NEXT born*) or infant* or babies))):ti,ab,kw	16790
#2	((preterm* or pre-term*) NEAR/2 (formula or nutrit*)):ti,ab,kw	402
#3	(necroti* NEAR/2 enterocolit*):ti,ab,kw	1505
#4	((neonat* or neo-nat* or new*borns or (new* NEXT born*) or babies) NEAR/3 (septicem* or septicaem* or sepsis or infect*)):ti,ab,kw	1315
#5	#1 or #2 or #3 or #4	17840
#6	(pregestat* or preconcept* or conception or prepregnan* or pregnan* or mid-gestat* or midgestat* or gravidity or multigravid* or multigestat* or (during NEXT gestation) or placenta* or (preterm NEXT ruptur*) or (preterm NEXT birth*) or (preterm NEXT labor) or (preterm NEXT labour) or PPROM or PROM or fetal or fetus* or foetal or foetus* or antenat* or ante-nat* or prenat* or pre-nat* or antepart* or ante-part* or peripart* or peri-part* or postpart* or post-part* or perinat* or peri-nat* or pregestat* or preeclamp* or eclamp* or HELPP or ((maternal or gestation*) NEAR/3 (diabet* or hyperten*)) or HIV* or AIDS or tuberculos* or ((mother* or women or matern*) not (infant* or neonat* or neo-nat* or (new* NEXT born*) or new*born*)) or ((parenteral* or intravenous* or intra-venous* or iv) not (enteral* or enteric or oral*))):ti or (((maternal or mother* or women or antenat* or ante-nat* or prenat* or pre-nat*) NEAR/6 (suppl* or diet* or nutrition)) not ((infant* or neonat* or neo-nat* or newborn* or (new* NEXT born*)) NEAR/6 (suppl* or diet* or nutrition))):ti,ab,kw	113085
#7	#5 not #6	14492
#8	(((supplementat* or supplementing or supplemented) not (suppl* NEAR/3 oxygen*)) or unsupplement* or un-supplem* or nonsupplem* or non-supplem* or ((diet* or nutri* or enteral* or enteric or oral* or os or oro* or nasogastr* or experimental or enhanc* or enrich* or intake or added or contain* or whey or milk or breastmilk or formula or formulas or powder) NEAR/2 (supplements or supplement))):ti,ab,kw	48147
#9	#7 and #8	1328
#10	#9 not (clinicaltrials or trialsearch):so	1109
#11	#10 with Publication Year from 2000 to 2020, in Trials	782

Part 2

#	Searches	Results
#1	(((prematur* or pre-matur* or immatur* or preterm* or pre-term* or VLBW* or ELBW* or LBW or low-birth-weight) near/6 (neo-nat* or neonat* or newborn* or born* or infant* or babies or child* or pediatr* or paediatr*)) or prematurity or ((SGA or small-for-gestational-age) near/6 (neo-nat* or neonat* or newborn* or (new* NEXT born*) or infant* or babies))):ti,ab,kw	16790
#2	((preterm* or pre-term*) NEAR/2 (formula or nutrit*)):ti,ab,kw	402
#3	(necroti* NEAR/2 enterocolit*):ti,ab,kw	1505
#4	((neonat* or neo-nat* or new*borns or (new* NEXT born*) or babies) NEAR/3 (septicem* or septicaem* or sepsis or infect*)):ti,ab,kw	1315
#5	#1 or #2 or #3 or #4	17840
#6	(pregestat* or preconcept* or conception or prepregnan* or pregnan* or mid-gestat* or midgestat* or gravidity or multigravid* or multigestat* or (during NEXT gestation) or placenta* or (preterm NEXT ruptur*) or (preterm NEXT birth*) or (preterm NEXT labor) or (preterm NEXT labour) or PPROM or PROM or fetal or fetus* or foetal or foetus* or antenat* or ante-nat* or prenat* or pre-nat* or antepart* or ante-part* or peripart* or peri-part* or postpart* or post-part* or perinat* or peri-nat* or pregestat* or preeclamp* or eclamp* or HELPP or ((maternal or gestation*) NEAR/3 (diabet* or hyperten*)) or HIV* or AIDS or tuberculos* or ((mother* or women or matern*) not (infant* or neonat* or neo-nat* or (new* NEXT born*) or new*born*)) or ((parenteral* or intravenous* or intra-venous* or iv) not (enteral* or enteric or oral*))):ti or (((maternal or mother* or women or antenat* or ante-nat* or prenat* or pre-nat*) NEAR/6 (suppl* or diet* or nutrition)) not ((infant* or neonat* or neo-nat* or newborn* or (new* NEXT born*)) NEAR/6 (suppl* or diet* or nutrition))):ti,ab,kw	113085
#7	#5 not #6	14492
#8	(((added or adding or additions) NEAR/6 (formula* or powder or milk or breastmilk or enteral* or enteric or feed* or nutrition or diet or diets)) or ((experimental or content* or composit* or free or deplet*) NEAR/2 formula*) or (formula* NEXT containing) or (formula* NEXT without)):ti,ab,kw	3425
#9	(((enteral* or enteric) NEAR/2 (administ* or enrich* or dose or doses or daily)) or ((enteral* or enteric) NEAR/1 (given or intervent*))):ti,ab,kw	783
#10	((oral* or os or oro* or nasogastr* or intragastric or intra-gastr* or enteral* or enteric or fortif* or enrich* or suppl* or replac*) NEAR/6 (enzym* or (amino NEXT acid*) or aminoacid* or antioxidant* or anti-oxidant* or (bioactiv* NEXT factors))):ti,ab,kw	4826
#11	(prebiot* or pre-biot* or synbiot* or syn-biot* or oligosacch* or oligo-sacch* or fructooligo* or galactooligo* or xylooligo* or HMO or HMOs or SCGOS* or LCFOS* or GOS or FOS or inulin* or fructan* or galactan* or pectin* or AOS or pAOS or betaglucan* or glucan*):ti,ab,kw	5666
#12	(((bovine or oro* or oral* or buccal* or enteral* or enteric) NEAR/6 colostrum) or forticolos or precolos):ti,ab,kw	178
#13	(whey or casein* or gl*comacropeptid* or gl*co-macropept* or GMP or lact*albumin* or lacto-albumin* or lact*ferrin* or lacto-ferrin* or bLF or bovine-LF or TLF or LIFT or ELFIN or Talactoferrin* or lactadherin* or MFGM* or lactogammaglobulin* or lacto-gammaglobulin* or milk-fat-globulin-membran*):ti,ab,kw	5724
#14	(caroten* or betacarot* or lycopen* or xanthophyl* or lutein or zeaxanthin* or zea-xanthin* or cryptoxanthin* or crypto-xanthin* or zeaxantin* or zea-xantin* or cryptoxantin* or crypto-xantin*):ti,ab,kw	3273
#15	(vitamin-A or vit-A or vitamin-C or vit-C or vitamin-E or vit-E):ti,ab,kw	9053

#16	((oral* or os or oro* or nasogastr* or intragastric or intra-gastr* or enteral* or enteric or fortif* or enrich* or suppl* or replac*) NEAR/6 (vit or vitamin* or multivitamin* or retinol or ascorbic* or ascorbat* or tocopherol* or alphatocopherol* or tocotrienol*)):ti,ab,kw	13717
#17	((oral* or os or oro* or nasogastr* or enteral* or enteric or fed or feed* or nutrition* or diet or diets or formula or formulas or whey or fortif* or enrich* or added or adding or additions or suppl* or mix*) NEAR/6 ((trace NEXT element*) or multimicronutrien* or micronutrient* or zinc or Zn* or selen* or copper or mangan* or molybden* or iodine)):ti,ab,kw	5006
#18	(acetylcysteine or Nacetylcysteine or NAC or cysteine or cystine or ((oral* or os or oro* or nasogastr* or enteral* or enteric or fed or feed* or nutrition* or diet or diets or formula or formulas or powder or whey or fortif* or enrich* or added or adding or additions or administration or dose or doses or content or composit* or contain* or suppl* or mix*) NEAR/6 ((essential NEXT amino NEXT acid*) or arginin* or histidin* or isoleucin* or leucin* or (lysin* not (ibuprof* NEAR/3 lysin*)) or (methionin* not (adenosyl NEAR/2 methionin*)) or phenylalanin* or fenylalanin* or threonin* or phosphothreonin* or phosphotreonin* or tryptophan* or tyrosine or valine or asparagine or glutamin* or glycine or levocarnitin* or carnitin*))):ti,ab,kw	10512
#19	((recomb* NEAR/10 lipase*) or r-lipas* or rh-lipas* or bucelipas* or BSSL* or rhBSSL* or ((oral* or os or oro* or nasogastr* or enteral* or enteric or fed or feed* or nutrition* or diet or diets or formula or formulas or powder or whey or fortif* or enrich* or added or adding or additions or administration or dose or doses or content or composit* or contain* or suppl* or mix*) NEAR/6 (lipas* or esteras* or carboxylesteras* or glycosidas* or protease* or amylas* or acetylhydrolas* or (acetyl NEAR/3 hydrolas*) or esteras* or catalas* or (superoxide NEXT dismutase*) or rhSOD* or CuZNSOD* or SOD or reductas* or oxi*reductas* or peroxidas* or lact*peroxida* or transferas* or lysozym* or muramidas*))):ti,ab,kw	3280
#20	(polyunsaturat* or (poly NEXT unsaturat*) or monounsaturat* or (mono NEXT unsaturat*) or MUFA or MUFAs or PUFA or PUFAs or LCPUFA* or LCP or LCPs or docosahex* or DHA or eicosapent* or icosapent* or EPA or omega-3* or omega-6* or omega3* or omega6*):ti,ab,kw or ((n3 or n6 or "n-3" or "n-6"):ti,ab,kw and ((fatty NEXT acid*) or LA or ALA)):ti,ab,kw	11278
#21	(linolenic or linolenate* or alphalinolen* or gammalinolen* or GLA or DGLA or arachidon* or ARA):ti,ab,kw not (cutan* or transcutan* or subcutan*).ti.	12717
#22	((oral* or os or oro* or nasogastr* or enteral* or enteric or fed or feed* or nutrition* or diet or diets or formula or formulas or powder or whey or fortif* or enrich* or added or adding or additions or content or composit* or contain* or suppl* or mix*) NEAR/6 (fatty NEXT acid*)):ti,ab,kw not (cutan* or transcutan* or subcutan*).ti.	13204
#23	(melatonin* or levothyroxin* or levoxin* or levo-thyroxin* or (L-thyroxin*) or LT4 or "LT-4"):ti,ab,kw	3572
#24	#8 or #9 or #10 or #11 or #12 or #13 or #14 or #15 or #16 or #17 or #18 or #19 or #20 or #21 or #22 or #23	78268
#25	#7 and #24	1724
#26	#25 not (clinicaltrials or trialsearch):so	1414
#27	#26 with Publication Year from 2000 to 2020, in Trials	819

Part 3

#	Searches	Results
#7	#5 not #6	14492
#8	(((oral* not (oral NEXT glucos*)) or os or oro* or nasogastr* or intragastric or (intra NEXT gastr*) or enteral* or enteric or fortif* or enrich* or suppl* or replac*) NEAR/9 (recombinant or hormon* or cytokin* or chemokin* or GLP or glucagon* or GLP1 or GLP2 or PYY or (peptid* NEXT YY) or leptin* or adipokin* or adiponect* or ADPN or adipofibrokin* or adipocytokin* or adipoq* or resistin* or FIZZ3 or (FIZZ NEXT 3) or (inflammator* NEAR/4 protein*) or ADSF or RELM or visfatin* or VISF or NAMPT* or NAmPRTas* or ((NMN or nicotinamid* or pyrophosphat*) NEAR/2 (pyrophosphorylas* or phosphoribosyltransferas* or transphosphoribosylas*)) or PBEF or PBEF1 or (colony NEXT enhanc* NEXT factor*) or obestatin* or ghrelin* or Ppghrelin* or apelin* or APJ or "APE-12" or APE12 or "APE-13" or APE13 or "APE-36" or APE36 or APLN* or estradiol* or oestradiol* or estriol or oestriol or estrogen* or oestrogen* or estron* or progest* or hydroxyprogest* or calcitonin* or calcitrin* or somatostatin* or prolactin* or cortisol or cortisone or hydrocortison* or thyro* or T4 or "T-4" or liothyronin* or T3 or "T-3" or insulin* or (carrier NEXT protein*) or (binding NEXT protein*) or nucleobindin* or NUCB2 or nesfatin* or NEFA or IGFB* or (calcium NEXT binding) or calprotectin* or calgranulin* or 27E10 or (CABP NEXT (P8 or P14)) or MRP8 or "MRP-8" or 60B8* or MRP14 or "MRP-14" or S100* or S-100A* or S-100B* or dermatopontin* or (dpt NEAR/3 protein) or discoidin* or (growth NEXT factor*) or somatomedin* or IGF or IGF1 or IGF2 or IGFB* or rhIGF* or EGF or urogastron* or TGF* or HGF or hepatopoietin* or neurotrophic factor* or NGF* or BDNF* or GDN* or VEGF* or sVEGF* or vasculotropin* or ANG or ANG1 or ANG2 or angiopoietin* or FGF* or colony-stimulating-factor* or CSF* or GCSF* or GMCSF* or EPO or rhEPO or rHuEPO or HuEPO or erythropoietin* or epoietin* or darpoetin* or interleukin* or lymphokin* or IL or IL7 or IL8 or CXCL* or "C-X-C" or CXC or "MCP-1" or "MCP1" or sCXCL* or IL10 or "CSIF-10" or CSIF10 or TNF* or ((tumor or tumour) NEAR/2 necrosis NEXT factor*) or MIF or (migration NEXT inhibition NEXT factor*) or immunoglob* or Ig or IgA or sIgA or IgG* or IgM*)):ti,ab,kw	28273
#9	#7 and #8	318
#10	#9 not (clinicaltrials or trialsearch):so<br>with Publication Year from 2000 to 2020, in Trials	198

Table S2. Overview of ongoing or recently completed studies investigating the effect of either bovine colostrum or lactoferrin

Intervention group (dosage)	Control group	Duration of the intervention	Estimated number of participants	Main inclusion criteria	Country	Year of registration	Trial registry	Trial registry identifier
				**Bovine colostrum**				
Bovine colostrum when own mother's milk is not or in no sufficient amounts available	Preterm formula if own mother's milk is not or in no sufficient amounts available	First 2 weeks of life	350	GA 26+0-31+6 weeks	China	2017	ClinicalTrials.gov	NCT03085277
Bovine colostrum as a fortifier to human milk	PreNAN FM 85 as fortifier to human milk	Until 35+6 weeks PMA or in no-need of fortification due to sufficient growth, whichever comes first	136	GA 26+0-30+6	China	2019	ClinicalTrials.gov	NCT03822104
Bovine colostrum as a fortifier to human milk	PreNAN FM 85 as fortifier to human milk	Until 34+6 PMA, discharge to another hospital or discharge to home	200	GA 26+0-30+6	Denmark	2018	ClinicalTrials.gov	NCT03537365
Bovine colostrum	Preterm formula	First 2 weeks of life	80	GA ≤34 weeks	Egypt	2019	ClinicalTrials.gov	NCT03926390
				**Lactoferrin**				
Lactoferrin (100 mg/day)	Standard care	Not reported	1300	GA ≤32 weeks and/or BW ≤1500 g	Italy	2010	ClinicalTrials.gov	NCT01172236
Bovine lactoferrin (200 mg/day)	Placebo	Until discharge	60	GA <32 weeks, BW <1500 g	Turkey	2011	ClinicalTrials.gov	NCT01287507
Lactoferrin (group 1: 100 mg/day, group 2: 150 mg/kg twice daily)	Placebo	Not reported	180	GA ≤36 weeks, BW 500-2500 g	Egypt	2013	ClinicalTrials.gov	NCT01821989
Bovine lactoferrin (group 1: 100 mg/day, group 2: 100 mg/day)	Placebo	4-6 weeks	180	GA <37 weeks	Egypt	2016	ClinicalTrials.gov	NCT02959229
Bovine lactoferrin (group 1: 150 mg/day, group 2: 300 mg/day)	Placebo	1 month	300	GA 28+0-36+6, BW 1000-2500 g	Pakistan	2018	ClinicalTrials.gov	NCT03431558
Group 1: standard formula with lactoferrin (1 mg/100 mL), group 2: standard formula with galacto-oligosaccharides	Standard formula	Start: first week of life, duration: until 6 weeks after the infants achieved full enteral feeding	60	GA 26+0-25+6	The Netherlands	2007	ISRCTN registry	ISRCTN71737811
Bovine lactoferrin (200 mg/kg/d) (1)*	Placebo	Until 34 weeks PMA or for a mimimum of 2 weeks, whichever is longer, or until discharge home or transfer to a secondary hospital, if earlier.	500	BW <1500 g	Canada	2017	ClinicalTrials.gov	NCT03367013

*Similar study protocol as used in the LIFT_ANZ study (2). The enrolled infants will be added to the 1542 infants already enrolled in the LIFT_ANZ study. GA: gestational age, BW: birth weight, PMA: postmenstrual age.

References

1. Asztalos EV, Barrington K, Lodha A, Tarnow-Mordi W, Martin A. Lactoferrin infant feeding trial_Canada (LIFT_Canada): protocol for a randomized trial of adding lactoferrin to feeds of very-low-birth-weight preterm infants. BMC Pediatr. 2020;20(1):40.
2. Tarnow-Mordi WO, Abdel-Latif ME, Martin A, Pammi M, Robledo K, Manzoni P, et al. The effect of lactoferrin supplementation on death or major morbidity in very low birthweight infants (LIFT): a multicentre, double-blind, randomised controlled trial. The Lancet Child & Adolescent Health. 2020;4(6):444-54.
